# Incidence and Risk Factors for Perioperative Cardiovascular Complications in Spine Surgery

**DOI:** 10.12688/f1000research.75245.2

**Published:** 2022-03-17

**Authors:** Haruthai Chotisukarat, Phuping Akavipat, Pathomporn Suchartwatnachai, Pimwan Sookplung, Jatuporn Eiamcharoenwit

**Affiliations:** 1 Department of Anesthesiology, Neurological Institute of Thailand, Bangkok, 10400, Thailand

**Keywords:** perioperative complication, cardiac arrest, myocardial infarction, congestive heart failure

## Abstract

**Background: **An increasing number of patients are opting for spine surgery despite the associated risk of cardiovascular complications. The evidence regarding the incidence and risk factors of cardiovascular complications in spine surgery is insufficient. Therefore, we aimed to determine the incidence and risk factors for cardiovascular complications that occur perioperatively in spine surgery.

**Methods: **This retrospective study included all patients who underwent spine surgery between January 2018 and December 2019 at a single center. Demographic, clinical, and operative data were collected from electronic medical records. The incidence of perioperative cardiac complications was determined. Univariate and multivariate analyses were performed to identify risk factors for the development of perioperative cardiovascular complications in the participants.

**Results: **Of the 1,002 eligible patients enrolled in the study, six developed cardiac complications. Acute myocardial infarction, cardiac arrest, and congestive heart failure occurred in one, two, and three patients, respectively. Risk factors for cardiovascular complications included scoliosis surgery (odds ratios (OR): 18.61; 95% confidence interval (CI): 1.346-257.35) and a history of congestive heart failure (OR: 120.97; 95% CI: 2.12-6898.80).

**Conclusion: **The incidence of perioperative cardiovascular complications in patients who underwent spine surgery was 0.6%. High-risk patients should be closely monitored optimally managed throughout the perioperative period.

## Introduction

The number of patients undergoing spine surgery tends to increase every year. ^1^^,^^2^ Approximately 900,000 spine surgeries are performed yearly in the United States, and the mean age of patients tends to increase every subsequent year. ^1^ A Japanese study found that the average age for degenerative spine surgery was 54.6 years in 2004 and increased to 63.7 years in 2015. ^2^ This is a cause for concern because older patients are predisposed to cardiovascular complication. Spine surgery often extends over a long operative duration and is likely to result in substantial intraoperative blood loss.

Currently, risk assessment of perioperative cardiovascular complications follows the 2014 ACC/AHA guidelines. ^3^ The revised cardiac risk index (RCRI) is a widely accepted tool for determining the risk of cardiovascular complications preoperatively. ^4^ However, the applicability of these guidelines and tools is limited in emergency surgery and different types of spine surgery. Although several studies worldwide have explored the risk factors of cardiovascular complications in patients undergoing spine surgery, ^5^^–^^7^ they do not inform regarding the role of intraoperative hypertension, hypotension, or blood loss. Hallqvist et al. found that hypotension during surgery can cause ischemic heart disease during the perioperative period. ^8^ Identifying the role of such intraoperative factors may help reduce the incidence of cardiovascular complications in spine surgery. Therefore, the objectives of this study are to examine the incidence and risk factors, including intraoperative hypertension, hypotension, and blood loss, of cardiovascular complications in spine surgery.

## Methods

### Study design

This retrospective cohort study was conducted after approval from the Research Ethics Committee of the Neurological Institute of Thailand (approval number IRB63040). Data were collected from patients who underwent spine surgery in a single hospital at the Neurological Institute of Thailand. The sample size was calculated by estimating an infinite population proportion with n4studies software (Ngamjaras C. et al., The Thailand Research Fund, Prince of Songkla University, Songkla, Thailand, 2016). Based on a study by Chalacheewa et al. wherein the configuration of error (d) of 0.01, the incidence of cardiovascular complications after anesthesia in older patients undergoing orthopedic surgery was 2.3%. ^9^ Using this information, and considering a dropout rate of 20%, a sample size of 863 patients was determined for our study. The final sample comprised 1,035 patients.

Data for a period of two years, between January 2018 and December 2019, were collected from inpatient medical records and an electronic anesthesia recording system. Patient data included demographic characteristics, the American Society of Anesthesiologists (ASA) physical status classification, laboratory findings, surgical data, and anesthesia-related parameters such as intraoperative blood pressure and amount of blood loss.

### Terminology

Cardiovascular complications including cardiac arrest, acute congestive heart failure (CHF), acute myocardial infarction (MI), and acute stroke were defined according to the following definitions of major adverse cardiac events (MACEs) ^10^:

1. Cardiac arrest was an abrupt loss of heart function, breathing, and consciousness that needed treatment with resuscitation, electric shock, or inotropic drugs. ^11^ Intraoperative cardiac arrest (IOCA) was cardiac arrest occurring in the operating room from the induction of anesthesia until the termination of anesthesia. The basis for the judgment of IOCA was whether the electrocardiogram (ECG) showed ventricular fibrillation, sudden disappearance of direct arterial blood pressure, and reduction of mean arterial pressure (MAP) to less than 20 mmHg. ^25^
2. Acute CHF was the rapid development of signs and symptoms of heart failure, diagnosed by the presentation of a new S3 gallop, jugular venous distension, rales sound in lung, and pulmonary edema or pleural effusion in chest X-ray (CXR). ^12^
3. Acute MI referred to myocardial necrosis resulting from impaired blood flow to the myocardium (Type I) or an imbalance between myocardial oxygen supply and demand (Type II). New elevation in troponin levels higher than the 99th percentile of the upper reference limit (UNL) included at least one of the following features: ischemic nature of the chest pain, recent significant electrocardiography (ECG) findings such as ST- segment or T-wave alterations, left bundle branch block or the presence of Q waves, and new-onset regional wall motion abnormalities (RWMA) on echocardiography. ^13^
4. Acute stroke was an episode of acute neurological dysfunction presumed to be caused by ischemia or hemorrhage that persisted for over 24 hours or caused death. ^14^
5. Intraoperative hypertension was defined as an increase in systolic blood pressure (SBP) greater than 20% from baseline for longer than 5 min. ^15^
6. Intraoperative hypotension was defined as SBP <100 mmHg or a reduction of SBP greater than 30% from baseline for more than 5 min. ^16^
7. Abnormal ECG findings included bradyarrhythmia, tachyarrhythmia, premature contractions, ST-segment deviations, T-wave inversion, or Q-wave presentation. ^17^
8. Abnormal CXR findings referred to abnormalities, such as infiltration, mass, water, air, effusion, lung atelectasis, and cardiomegaly in chest radiography of the lung or heart. ^18^
9. Anemia was defined as blood hemoglobin levels lower than sex-specific standards, i.e., <12.0 g/dL in women and <13.0 g/dL in men. ^19^
10. Scoliosis surgery referred to surgery performed to treat adult degenerative scoliosis. The technique undertaken for scoliosis surgery varied based on disease severity and included decompression alone, decompression with short-segment fusion, or decompression coupled with long fusion and correction of the deformity. ^20^
11. Intraoperative blood transfusion was defined as transfusion of red blood cell to the patient during the surgery. The criteria for transfusion were reduction in hemoglobin concentrations to 7–10 g/dL, risk or occurrence of continuous bleeding, intravascular volume depletion or development of any signs of organ ischemia, and inadequate cardiopulmonary reserve. ^21^

Only MACEs that occurred perioperatively and within 30 days postoperatively were included as cardiovascular complications in this study.

### Statistical analysis

SPSS IMB Version 22 (IBM Corporation, New York, USA, 2013) was used for the data analysis. Descriptive statistics were used and presented as numbers, percentages, and means ± standard deviations. Logistic regression was used to identify the cardiovascular risk factors. Fisher's exact test was used to evaluate the association between each categorical variable and cardiovascular complications. The association between each continuous variable and cardiovascular complications was evaluated using unpaired t-tests. Multivariate log-binomial regression was used to determine the association between each risk factor and cardiovascular complications. Risk factors were included in the multivariate log-binomial regression model if their univariate association had a p-value <0.2. The results are presented as p-values, odds ratios (ORs), adjusted ORs, and 95% confidence intervals (CIs). A p-value < 0.05 was considered statistically significant.

## Results

A total of 1,035 patients who underwent spine surgery were included. On exclusion of 33 patients with incomplete data, 1,002 patients remained, of which 550 (55%) were women and 452 (45%) were men. The mean age was 60 ± 12 years, and mean body mass index (BMI) was 25.41 ± 4.3 kg/m ^2^. The most common surgical interventions were posterior lumbar fusion (40.7%) and anterior cervical discectomy with fusion (23.8%). Patient demographics, surgical factors, and anesthesia factors are shown in Table 1.

**Table 1.** Patient demographics, surgical factors, and anesthesia factors.

Variables	Number	Percent
Sex:		
Female	550	55
Male	452	45
ASA physical status:		
I	91	9.1
II	610	60.9
III	299	29.8
IV	2	0.2
Laboratory and investigation:		
Hemoglobin <12 g/dL	147	14.7
Creatinine clearance < 60	67	6.7
Abnormal ECG	230	23
Abnormal CXR	155	15.5
Underlying disease:		
Diabetes mellitus	208	20.8
Hypertension	524	52.4
Chronic kidney disease	71	7.1
Stroke	41	4.1
Obstructive sleep apnea	98	9.8
Thyroid disease	34	3.4
Chronic pulmonary disease	40	4.0
Cardiac arrhythmia	29	2.9
Congestive heart failure	2	0.2
Myocardial infarction	43	4.3
Surgical condition:		
Emergency	29	2.9
Elective	973	97.1
Surgical interventions:		
Anterior cervical discectomy and fusion (ACDF)	238	23.8
Posterior cervical fusion	43	4.3
Posterior cervical decompression	16	1.6
Posterior lumbar decompression	48	4.8
Posterior lumbar fusion	408	40.7
Discectomy	92	9.2
Scoliosis surgery	36	3.6
Spinal cord tumor surgery	93	9.3
Others	28	2.8
Number of fusion levels:		
1-2 level	657	65.6
3-4 level	276	27.5
>4 level	69	6.9
Intraoperative events:		
Hypotension	215	21.5
Hypertension	80	8.0
Blood transfusion	157	15.7

ASA, American Society of Anesthesiologists; ECG, electrocardiography; CXR, chest X-ray.

All patients underwent surgery under general anesthesia. Six patients, three men and three women, with a mean age of 65.67 ± 7.8 years (p = 0.235), developed cardiovascular complications (0.6%). The incidence was higher in the group without cardiovascular complications, which had a mean age of 59.53 ± 12.7 years. The mean BMI of patients who developed cardiovascular complications was 24 ± 4.89 kg/m ^2^ (p = 0.434).

Of the six cardiovascular complications that occurred in our sample, two (one cardiac arrest, one acute CHF) developed intraoperatively and four postoperatively (one cardiac arrest, three acute CHF). Five out of six complications occurred during elective surgery (two scoliosis surgeries, one posterior cervical fusion, two posterior lumbar fusion) and only one during an emergency surgery (laminectomy with blood clot removal).

We found that three patients with acute CHF and one with acute MI had substantial blood loss during the operation (700-3,000 mL) and prolonged operation time (173-375 min). Airway obstruction was found as a potential cause for postoperative cardiac arrest. The patient who experienced a cardiac arrest intraoperatively was a 72-year-old man without any underlying disease but with an abnormal ECG finding of a premature atrial contraction immediately prior to surgery. Posterior lumbar fusion was performed for this patient at one level. At 54 min after surgery, he experienced a cardiac arrest. Cardio Pulmonary Resuscitation (CPR) was performed for 5 min, after which spontaneous circulation was re-established. The diagnosis of this condition was acute MI.

Univariate analysis revealed that a history of CHF before spine surgery was statistically significant with incidence of cardiovascular complications ( Table 2), and the median amount of intraoperative blood loss, which was 1,000 mL in the cardiovascular complication group and 250 mL in the non-cardiovascular complication group (p = 0.046).

**Table 2.** Univariate Analysis of the risk for cardiovascular complications in spine surgery.

Variables	Without cardiovascular complication	With cardiovascular complication	Odd ratio	95% confidence interval	p-value
Sex:					
Female	547	3			
Male	449	3	1.21	0.245-6.07	1
Surgical condition:					
Elective	968	5			
Emergency	28	1	6.91	0.78-61.14	0.162
ASA physical status:					
I-II	697	4			
III-IV	299	2	1.16	0.21-6.39	1
Laboratory and investigation:					
Hemoglobin < 12 g/dL	145	2	2.93	0.53-16.17	0.215
Creatinine clearance <60	66	1	2.82	0.32-24.47	0.341
Underlying disease:					
Diabetes Mellitus	206	2	1.92	0.34-105	0.61
Hypertension	521	3	0.908	0.182-4.5	1
Myocardial infarction	42	1	4.54	0.52-39.75	0.23
Congestive heart failure	1	1	198	10.85-364	0.012 *
Surgical interventions:					
Scoliosis surgery	34	2	14	2.50-79.91	0.017 *
Intraoperative events:					
Hypotension	213	2	1.84	0.33-10.10	0.614
Hypertension	79	1	2.32	0.27-20.17	0.394
Blood transfusion	154	3	5.47	1.09-27.33	0.053

ASA: American Society of Anesthesiologists.

* Statistical significance at p < 0.05.

Multivariate analysis found that a history of CHF (OR 120.97; 95% CI, 2.12-6898.8) and scoliosis surgery (OR 18.61; 95%CI, 1.34-257.35) were risk factors associated with development of cardiovascular complications in patients who underwent spine surgery ( Table 3).

**Table 3.** Multivariate analysis of the risk for cardiovascular complications in spine surgery.

Variables	Adjusted odds ratio	95% Confidence interval	p-value
Emergency surgical condition	4.65	0.179-121.28	0.355
Scoliosis Surgery	18.61	1.346-257.35	0.029 *
Hemoglobin < 12 g/dL	2.02	0.184-22.02	0.566
Congestive heart failure	120.97	2.12-6898.80	0.02 *
Amount of blood loss (mL)	1.000	0.999-1.001	0.99

* Statistical significance at p < 0.05.

## Discussion

Most patients who undergo spine surgery are older adults and are predisposed to physiological changes in the circulatory system, including loss of elasticity of blood vessels leading to high blood pressure. In addition, older adults may have other comorbidities, such as diabetes and kidney disease. Spine surgery usually has a high risk of blood loss, especially in older adults. Older adults are also more likely to develop cardiovascular complications. The incidence (0.6%) of cardiovascular complications noted in patients undergoing spine surgery at our institution was within the range (0.13-1.6%) observed in previous studies. The width of the range may differ according to the duration of the postoperative data collection and definitions. For example, we defined a cardiovascular complication as any MACE that occur intraoperatively until 30 days postoperatively, while other studies only included cardiac arrest and acute MI in the definition. ^5^

We found that patients with a history of CHF before surgery had a high risk of cardiovascular complications. Chalacheewa et al. found that older patients with a history of CHF had a significantly greater risk of incident cardiovascular complications in orthopedic surgery. ^9^ Similarly, Bovonratwet et al. found that older patients with a history of heart failure had a significantly high mortality rate within 30 days after spine surgery. ^5^ Preoperative diastolic dysfunction in patients with a history of CHF was a possible etiology. These patients showed a reduction in the threshold of hypovolemic tolerance. Additionally, spine surgery is likely to result in massive blood loss, which often leads to significant hypotension and, consequently, hypervolemia that causes an exaggerated increase in left atrial pressure, leading to pulmonary edema. ^22^

In this study, almost all scoliosis surgeries were performed to treat degenerative scoliosis. Surgical intervention included decompression alone and fusion of more than three levels. Passia et al reported that scoliosis surgery is a significant risk factor for cardiovascular complications. ^23^ However, Bovonratwet et al found that the type of spine surgery is not a risk factor because almost all spine surgeries involve anterior lumbar procedures (67.76%). ^5^ In contrast, most spine surgeries performed in our neurological institution involved posterior lumbar procedures (45.5%).

Our findings are contrary to Hallqvist et al’s who reported that intraoperative hypotension is not a risk factor for cardiovascular complications. ^8^ A possible explanation for this discrepancy could the difference in definition of intraoperative hypotension. In our study, intraoperative hypotension was defined as SBP less than 100 mmHg or a 30% reduction of SBP from baseline for at least 5 min, whereas Hallqvist et al. defined it as a reduction of SBP by 20 mmHg or more for at least 5 min. ^8^

The tools used to calculate the cardiac risk index before surgery have many variations with different reliabilities and validities. The RCRI or Lee Index ^4^ is used to calculate the risk of cardiac complications before surgery and includes the following six valued scores: 1. High-risk surgery: intraperitoneal, intrathoracic, or vascular surgery; 2. history of heart disease; 3. history of CHF; 4. history of stroke; 5. history of insulin use; and 6. creatinine level > 2.0 mg/dL. Our findings corroborate the evidence from RCRI that history of CHF is a risk factor for cardiovascular complications in spine surgery. However, scoliosis surgery was not identified in the RCRI. According to the 2014 ACC/AHA guidelines, ^3^ spine surgery carries an intermediate risk. The American College of Surgeons NSQIP Surgical Risk Calculator, ^24^ identifies scoliosis surgery as a separate surgery type, and includes history of CHF as a risk factor for development of cardiovascular complications; therefore, it may be better suited for determining the risk of cardiovascular complication in spine surgery.

The design of this retrospective cohort study was limited by the quality of data collection and data completeness. For example, record of mean arterial pressure (MAP). A study with a prospective design is recommended.

In conclusion, the incidence of cardiovascular complications in spine surgery was 0.6%. The possible risk factors for these complications include a history of CHF before surgery and scoliosis surgery. Patients with these characteristics should be evaluated and the cardiac risk stratification should be optimized to provide these patients with special care intraoperatively and postoperatively to prevent complications during hospitalization.

### Data availability statement

Figshare. CVS risk_Raw Data_F1000 research.xlsx. DOI: https://doi.org/10.6084/m9.figshare.16923355. ^26^

Data are available under the terms of the Creative Commons Zero “No rights reserved” data waiver (CC BY 4.0 Public domain dedication).

### Grant information

Neurological Institute of Thailand, Bangkok. The grant number is 437219 and IRB63040

**Competing interests:** None

## References

[1] Cram P Landon BE Matelski J : Utilization and outcomes for spine surgery in the United States and Canada. *Spine.* 2019;44(19):1371–1380. 10.1097/BRS.0000000000003083 31261267 PMC6746582

[2] Kobayashi K Ando K Nishida Y : Epidemiological trends in spine surgery over 10 years in a multicenter database. *Eur Spine J.* 2018;27(8):1698–1703. 10.1007/s00586-018-5513-4 29435653

[3] Fleisher LA Fleischmann KE Auerbach AD : 2014 ACC/AHA guideline on perioperative cardiovascular evaluation and management of patients undergoing noncardiac surgery: executive summary: a report of the American College of Cardiology/American Heart Association Task Force on Practice Guidelines. *Circulation.* 2014;130(24):2215–2245. 10.1161/CIR.0000000000000105 25085962

[4] Bierle DM Raslau D Regan DW : Preoperative evaluation before noncardiac surgery. *Mayo Clin Proc.* 2020;95(4):807–822. 10.1016/j.mayocp.2019.04.029 31753535

[5] Bovonratwet P Bohl DD Malpani R : Cardiac complications related to spine surgery: timing, risk factors, and clinical effect. *J Am Acad Orthop Surg.* 2019;27(7):256–263. 10.5435/JAAOS-D-17-00650 30897607

[6] Fineberg SJ Oglesby M Patel AA : Incidence and mortality of perioperative cardiac events in cervical spine surgery. *Spine (Phila Pa 1976).* 2013;38(15):1268–1274. 10.1097/BRS.0b013e318290fdac 23486411

[7] Guyot JP Cizik A Bransford R : Risk factors for cardiac complications after spine surgery. *Evid-Based Spine Care J.* 2010;1(2):18–25. 10.1055/s-0028-1100910 23637663 PMC3623101

[8] Hallqvist L Granath F Fored M : Intraoperative hypotension and myocardial infarction development among high-risk patients undergoing noncardiac surgery: a nested case-control study. *Anesth Analg.* 2021;133(1):6–15. 10.1213/ANE.0000000000005391 33555690

[9] Chalacheewa T Sirinan C Apinyachon W : The incidence and risk factors of major cardiovascular complications. *Thai J Anesthesiol.* 2011;37(1):34–46.

[10] Miao B Hernandez AV Alberts MJ : Incidence and predictors of major adverse cardiovascular events in patients with established atherosclerotic disease or multiple risk factors. *J Am Heart Assoc.* 2020;9(2):e014402. 10.1161/JAHA.119.014402 31937196 PMC7033849

[11] Al-Khatib SM Stevenson WG Ackerman MJ : AHA/ACC/HRS Guideline for management of patients with ventricular arrhythmias and the prevention of sudden cardiac death. *Circulation.* 2018;138(13):e272–e391. 10.1161/CIR.0000000000000549 29084731

[12] Heart Failure Council of Thailand : *Heart failure guideline.* 3rd ed. Bangkok;2019.

[13] Magoon R Makhija N DasMagoon D : Perioperative myocardial injury and infarction following non-cardiac surgery: a review of the eclipsed epidemic. *Saudi J Anaesth.* 2020;14(1):91–99. 10.4103/sja.SJA49919 31998026 PMC6970380

[14] Sacco RL Kasner SE Broderick JP : An updated definition of stroke for the 21st century: a statement for healthcare professionals from the American Heart Association/American Stroke Association. *Stroke.* 2013;44(7):2064–2089. 10.1161/STR.0b013e318296aeca 23652265 PMC11078537

[15] Varon J Marik PE : Perioperative hypertension management. *Vasc Health Risk Manag.* 2008;4(3):615–627. 10.2147/VHRM.S2471 18827911 PMC2515421

[16] Kouz K Hoppe P Briesenick L : Intraoperative hypotension: pathophysiology, clinical relevance, and therapeutic approaches. *Indian J Anaesth.* 2020;64(2):90–96. 10.4103/ija.IJA_939_19 32139925 PMC7017666

[17] Wagner GS Macfarlane P Wellens H : AHA/ACCF/HRS Recommendations for the standardization and interpretation of the electrocardiogram. *Circulation.* 2009;119:262–270.

[18] Candemir S Antani S : A review on lung boundary detection in chest X-rays. *Int J Comput Assist Radiol Surg.* 2019;14(4):563–576. 10.1007/s11548-019-01917-1 30730032 PMC6420899

[19] Cappellini MD Motta I : Anemia in clinical-practice definition and classification: Does hemoglobin change with aging?. *Semin Hematol.* 2015;52(4):261–269. 10.1053/j.seminhematol.2015.07.006 26404438

[20] Cho KJ Kim YT Shin SH : Surgical treatment of adult degenerative scoliosis. *Asian Spine J.* 2014;8(3):371–381. 10.4184/asj.2014.8.3.371 24967054 PMC4068860

[21] Practice guidelines for perioperative blood management: an updated report by the American Society of Anesthesiologists Task Force on Perioperative Blood Management. *Anesthesiology.* 2015;122(2):241–275. 10.1097/ALN.0000000000000463 25545654

[22] Sellers D Srinivas C Djaiani G : Review article cardiovascular complications after non-cardiac surgery. *Anaesthesia.* 2018;73(1) Supplement 1:34–42. 10.1111/anae.14138 29313903

[23] Passias PG Poorman GW Delsole E : Adverse outcomes and prediction of cardiopulmonary complications in elective spine surgery. *Glob Spine J.* 2018;8(3):218–223. 10.1177/2192568217718817 29796368 PMC5958483

[24] Bilimoria KY Liu Y Paruch JL : Development and evaluation of the universal ACS NSQIP surgical risk calculator: a decision aid and informed consent tool for patients and surgeons. *J Am Coll Surg.* 2013;217(5):833–842.e3. 10.1016/j.jamcollsurg.2013.07.385 24055383 PMC3805776

[25] Han F Wang Y Dong J : Intraoperative cardiac arrest. *Medicine.* 2017;96(17):e6794. 10.1097/MD.0000000000006794 28445319 PMC5413284

[26] Chotisukarat H : CVS risk_Raw Data_F1000 research.xlsx. figshare. *Dataset.* 2021. 10.6084/m9.figshare.16923355.v1

