# Supplementary material for: Incidence and Risk Factors for Perioperative Cardiovascular Complications in Spine Surgery
Source: F1000Res. 2022 Mar 17;11:15. Originally published 2022 Jan 7. [Version 2] doi: 10.12688/f1000research.75245.2 (PMC8943344; doi:10.12688/f1000research.75245.2)
Supplement: Peer review history

# Peer review: Reviewer response for version 2

**DOI:** 10.5256/f1000research.122116.r127837

**Published:** 2022-03-23

**Authors:** Chanannait Paisansathan

**Affiliations:** 1 Department of Anesthesiology, University of Illinois College of Medicine, Chicago, IL, USA

I have no further comments to make.

Is the work clearly and accurately presented and does it cite the current literature?

Yes

If applicable, is the statistical analysis and its interpretation appropriate?

Yes

Are all the source data underlying the results available to ensure full reproducibility?

Yes

Is the study design appropriate and is the work technically sound?

Partly

Are the conclusions drawn adequately supported by the results?

Yes

Are sufficient details of methods and analysis provided to allow replication by others?

Yes

Reviewer Expertise:

Neuroanesthesia

I confirm that I have read this submission and believe that I have an appropriate level of expertise to confirm that it is of an acceptable scientific standard.

# Peer review: Reviewer response for version 2

**DOI:** 10.5256/f1000research.122116.r127838

**Published:** 2022-03-21

**Authors:** Rattaphol Seangrung

**Affiliations:** 1 Department of Anesthesiology, Faculty of Medicine Ramathibodi Hospital, Mahidol University, Bangkok, Thailand

Thank you for revising the article. However, I found the number of surgical conditions in the elective cases had some mistakes. It should be a total of 968. Please recheck it.

Is the work clearly and accurately presented and does it cite the current literature?

Partly

If applicable, is the statistical analysis and its interpretation appropriate?

Yes

Are all the source data underlying the results available to ensure full reproducibility?

Yes

Is the study design appropriate and is the work technically sound?

Yes

Are the conclusions drawn adequately supported by the results?

Yes

Are sufficient details of methods and analysis provided to allow replication by others?

Yes

Reviewer Expertise:

Anesthesiology and pain medicine.

I confirm that I have read this submission and believe that I have an appropriate level of expertise to confirm that it is of an acceptable scientific standard.

# Peer review: Reviewer response for version 1

**DOI:** 10.5256/f1000research.79092.r119101

**Published:** 2022-02-18

**Authors:** Rattaphol Seangrung

**Affiliations:** 1 Department of Anesthesiology, Faculty of Medicine Ramathibodi Hospital, Mahidol University, Bangkok, Thailand

Thank you for inviting me to review this article. Overall, this retrospective study demonstrated the major risk factors involved with intraoperative cardiac complications in spinal surgery, which is a piece of interesting data.

 For the question: Is the work clearly and accurately presented, citing the current literature?

- From *Table 1. Patient demographics, surgical factors, and anesthesia factors *and *Table 2. Univariate Analysis of the risk for cardiovascular complications in spine surgery*.
  - The author presented a number of variables: sex, surgical condition, underlying disease (hypertension, congestive heart failure) in Table 1 that differ from the sum of a total number of without and with cardiovascular complications in Table 2. That may affect the analysis process. Please clarify.
- From Table 2, the author presented that the total number of emergency surgery was 5, but in the results, the author described only one emergency patient had a cardiac complication; please clarify this.
- Intraoperative mean arterial pressure ****(MAP) below 60-70 mmHg is associated with myocardial injury in non-cardiac surgery. Why did the author not use MAP as the risk factor for predicting cardiac complications?

I would like to assign this article the status of Approved with reservations.

Is the work clearly and accurately presented and does it cite the current literature?

Partly

If applicable, is the statistical analysis and its interpretation appropriate?

Yes

Are all the source data underlying the results available to ensure full reproducibility?

Yes

Is the study design appropriate and is the work technically sound?

Yes

Are the conclusions drawn adequately supported by the results?

Yes

Are sufficient details of methods and analysis provided to allow replication by others?

Yes

Reviewer Expertise:

Anesthesiology and pain medicine.

I confirm that I have read this submission and believe that I have an appropriate level of expertise to confirm that it is of an acceptable scientific standard, however I have significant reservations, as outlined above.

# Response

**Published:** 2022-02-27

**Authors:** Haruthai Chotisukarat

**Affiliations:** Neurological Institute of Thailand, Thailand

**Title:** **Incidence of and risk factors for perioperative cardiovascular complications in spine surgery**

**Journal: F1000Research**

 Dear Dr. Rattaphol Seangrung,

 Thank you for giving us a chance to improve our research article. Hopefully, you would appreciate our revised version.

 Regards

 Haruthai Chotisukarat, Department of Anesthesiology, Neurological Institute of Thailand, Bangkok, Thailand

**Comments:**

- *From Table 1. Patient demographics, surgical factors, and anesthesia factors and Table 2. Univariate Analysis of the risk for cardiovascular complications in spine surgery. *
  - *The author presented a number of variables: sex, surgical condition, underlying disease (hypertension, congestive heart failure) in Table 1 that differ from the sum of a total number of without and with cardiovascular complications in Table 2. That may affect the analysis process. Please clarify.*
- *From Table 2, the author presented that the total number of emergency surgery was 5, but in the results, the author described only one emergency patient had a cardiac complication; please clarify this.*

**Response**: We have rechecked all the tables. Table 1 was found the incorrect part of the number of congestive heart failure. Table 2 was found that the incorrect part was the number of variables: sex, surgical condition, underlying disease (hypertension, congestive heart failure). Therefore, the correct table has been revised as attached here: Table 1 and Table 2.
- *Intraoperative mean arterial pressure ****(MAP) below 60-70 mmHg is associated with myocardial injury in non-cardiac surgery. Why did the author not use MAP as the risk factor for predicting cardiac complications? *
**Response:** In the practice of recording data for medication to increase blood pressure in our institution still use the systolic BP value, which is a limitation in this research (retrospective study). If there is a prospective study, it can record MAP and have complete data.

# Peer review: Reviewer response for version 1

**DOI:** 10.5256/f1000research.79092.r119103

**Published:** 2022-01-26

**Authors:** Chanannait Paisansathan

**Affiliations:** 1 Department of Anesthesiology, University of Illinois College of Medicine, Chicago, IL, USA

I would like to thank you for the invitation to review an article entitled “Incidence of and Risk Factors for Perioperative Cardiovascular Complications in Spine Surgery.” I have read this manuscript with enthusiasm. My comments are as follows:

1. The author could consider deleting “of” from the title. So, the new title will be “Incidence and Risk Factors for Perioperative Cardiovascular Complications in Spine Surgery.”
2. I noticed that authors are using the relative risk in the abstract. However, they report odds ratios in the results. Therefore, I suggest that the authors only use odds ratios to be consistent with the results.
3. On the study design, I am curious if the authors would be able to look at the mean arterial pressure (MAP) instead of only using the systolic blood pressure as the definition of intraoperative hypotension. Many articles which reported the association between intraoperative hypotension used both terms when investigating major adverse cardiac events (MACEs). (See Reference).
4. Table 2 clarifies the number (with cardiovascular complication) between Elective and Emergency conditions. I saw that both conditions have the same incidence of 5.

Thank you. I am looking forward to reading the revised version of this article.

Is the work clearly and accurately presented and does it cite the current literature?

Yes

If applicable, is the statistical analysis and its interpretation appropriate?

Yes

Are all the source data underlying the results available to ensure full reproducibility?

Yes

Is the study design appropriate and is the work technically sound?

Partly

Are the conclusions drawn adequately supported by the results?

Yes

Are sufficient details of methods and analysis provided to allow replication by others?

Yes

Reviewer Expertise:

Neuroanesthesia

I confirm that I have read this submission and believe that I have an appropriate level of expertise to confirm that it is of an acceptable scientific standard, however I have significant reservations, as outlined above.

## References

[1] : Intraoperative hypotension and the risk of postoperative adverse outcomes: a systematic review. *Br J Anaesth* .2018;121(4) : 10.1016/j.bja.2018.04.036 706-721 10.1016/j.bja.2018.04.036 30236233

# Response

**Published:** 2022-02-01

**Authors:** Haruthai Chotisukarat

**Affiliations:** Neurological Institute of Thailand, Thailand

**Title:** **Incidence of and risk factors for perioperative cardiovascular complications in spine** **surgery**

**Journal: F1000Research**

 Dear Dr. Chanannait Paisansathan,

 Thank you for giving a chance to improve our research article. Hopefully, you would appreciate our revised version.

 Regards,

 Haruthai Chotisukarat, 

 Department of Anesthesiology, Neurological Institute of Thailand, Bangkok, Thailand

**Comments:**

1. *The author could consider deleting “of” from the title. So, the new title will be “Incidence and Risk Factors for Perioperative Cardiovascular Complications in Spine Surgery.”*
**Response:** We will change the title to “Incidence and Risk Factors for Perioperative Cardiovascular Complications in Spine Surgery.”
2. *I noticed that authors are using the relative risk in the abstract. However, they report odds ratios in the results. Therefore, I suggest that the authors only use odds ratios to be consistent with the results.*
**Response:** We change the abstract in part to: **“**Results **: **Of the 1,002 eligible patients enrolled in the study, six developed cardiac complications. Acute myocardial infarction, cardiac arrest, and congestive heart failure occurred in one, two, and three patients, respectively. Risk factors for cardiovascular complications included scoliosis surgery (odds ratios [OR]:18.61; 95% confidence interval [CI]: 1.346-257.35) and a history of congestive heart failure (OR: 120.97; 95% CI: 2.12-6898.80).”
3. *On the study design, I am curious if the authors would be able to look at the mean arterial pressure (MAP) instead of only using the systolic blood pressure as the definition of intraoperative hypotension. Many articles which reported the association between intraoperative hypotension used both terms when investigating major adverse cardiac events (MACEs). (See Reference).*
**Response:  **We have reviewed the literature definitions applied to intraoperative hypotension as published in Anesthesiology by Bijker JB, et al. It can be integrated into clinical practice and appropriately used in the circumstances. The Royal College of Anesthesiologists of Thailand (RCAT) committee and we agree to use this definition for systolic blood pressure. Moreover, a lot of the literature uses this definition, e.g. Kouz K, et al.: Intraoperative hypotension: Pathophysiology, clinical relevance, and therapeutic approaches.
4. *Table 2 clarifies the number (with cardiovascular complication) between Elective and Emergency conditions. I saw that both conditions have the same incidence of 5.*
**Response:** We rechecked and found that the number of emergency were wrong. The correct number is 1. We change table 2 in light of this. Please see this link for the corrected table. 
